# Fractional microneedle radiofrequency for photoaging and atrophic acne scar: a split-face clinical observation of two devices

**DOI:** 10.1007/s10103-026-04946-w

**Published:** 2026-07-10

**Authors:** Yan Sun, Zi Qing Yu, Ying Zhang, Jin Xuan Wu, Miao Dong, Yuanyuan Xu, Xue Gang Xu, Xing Hua Gao, Shuai Qiao, Yan Wu

**Affiliations:** 1 Department of Dermatology, The First Hospital of China Medical University, Shenyang, China; 2 Department of Dermatology, Sheng Jing Hospital, Shenyang, China; 3 NHC Key Laboratory of Immunodermatology, Ministry of Education Key Laboratory of Immunodermatology, National Joint Engineering Research Center for Diagnosis and Treatment of Immunologic Skin Diseases, The First Hospital of China Medical University, Shenyang, China

**Keywords:** Fractional Microneedle Radiofrequency (FMR), Photoaging, Atrophic acne scar, Split-face, Clinical observation

## Abstract

Fractional microneedle radiofrequency (FMR) has been proven effective and safe for acne scar and skin rejuvenation, but the treatment outcomes vary across different FMR devices. The face of each participant was randomly assigned as treatment side (treated with the Peninsula Timerevert Needle) and control side (treated with the EndyMed) along the midline. Outcome measures include melanin index (MI), erythema index (EI), stratum corneum hydration (SCH), sebum and dermal density on all facial sites. The treatment duration, participant’s satisfaction score and physician’s global assessment (PGA) score, pain score, bleeding score, erythema score and other side effects were evaluated. A total of 20 participants completed the entire course. The participant’s satisfaction score and PGA score increased progressively over time. Compared with the control side, the treatment side exhibited a shorter treatment time and lower pain score, but higher bleeding score. Dermal density increased gradually following FMR treatment and was statistically significant higher at 3 months than that at other follow-up time points. There were no statistically significant differences between the treatment and control sides in participant’s satisfaction score, PGA score, erythema score, erythema duration and edema duration, MI, EI, SCH, sebum and dermal density at the forehead, cheek and chin of at any follow-up time point. FMR is effective and safe for skin photoaging and acne scar. Peninsula Timerevert Needle ^TM^ offers the advantages of less pain and shorter treatment duration comparing with EndyMed, but is associated with a higher incidence of bleeding.

## Introduction

Skin photoaging is a form of extrinsic skin aging which is characterized by wrinkles, telangiectasias, enlarged pores, roughness and hyperpigmentation in sun-exposed area, such as face and neck [1, 2]. It is primarily caused by chronic exposure to sunlight, including ultraviolet (UV) A and B [3]. Atrophic acne scar is a permanent complication of acne vulgaris [4], resulting from loss of collagen in the dermis [5]. Both photoaging and atrophic acne scar negatively impact patients physically, psychologically and socially, thereby leading to poor quality of life [6, 7]. Recently, they have drawn increasing concerns from dermatologists and patients.

Radiofrequency (RF) is a type of non-ionizing radiation, with the frequency ranging from 30 to 300 GHz. It locates in the low-energy end of the electromagnetic spectrum [8]. RF was initially described to treat back pain and neuralgias in the early 20th century, and later on it showed excellent efficacy in malignant cutaneous lesions, such as malignant melanoma, Kaposi sarcoma and mycosis fungoides [9]. Recently, researchers found that RF is a new method to improve skin texture, therefore, it has been applied to skin rejuvenation. It induces local hyperthermia, promotes the formation of new collagen in dermis, and thus remodels the connective tissue [10]. The electric fields were created by collisions between a pair of electrodes within a limited RF spectrum. The polar molecules vibrate 6 million times per second, resulting in controlled heating of target tissues [11]. RF devices are classified into monopolar, bipolar and multipolar systems. Monopolar RF devices transmit electromagnetic energy from a single active electrode toward a passive one. Monopolar RF systems create uniform volumetric heating, which are widely applied in skin laxity. However, patients always complain discomfort by monopolar RF compared with bipolar systems. The electrical current of bipolar RF devices is transferred between two positioned electrodes [12], which offers a more controlled and localized energy distribution. The penetration depth is approximately half the distance between the two electrodes. And patients suffer from limited discomfort during treatment. Multipolar RF devices cause hyperthermia by three or more electrodes, which prevent overheating and subsequent thermal injury to the target tissues [11]. Fractional microneedle radiofrequency (FMR) is a kind of radiofrequency with fractional microneedle electrodes, resulting in limited epidermal damage [13]. Recent studies have shown that FMR was effective and safe for acne scar [14] and skin rejuvenation [15]. There are several FMR devices manufactured by different companies in the market right now, but the advantages and limitations remain insufficiently characterized. Accordingly, we conducted this split-face clinical observation to compare the differences between 2 distinct multipolar FMR devices in treating photoaging and atrophic acne scar.

## Participants and Methods

### Participants

The study was performed from December 2023 to August 2024. Eligible participants were those attending outpatient clinic of Department of Dermatology, The First Hospital of China Medical University, with a clinical diagnosis of atrophic acne scar or photoaging. Participants were 18–60 years old, without gender restriction. Participants who were pregnant, lactate, had infection and/or inability to complete the whole process were excluded. Those with history of photosensitive diseases, keloid and/or severe systemic diseases were also excluded. Use of photosensitive drugs, retinoids, corticosteroids, immunosuppressants and/or anticoagulants in the previous month were forbidden. Before enrollment, each participant voluntarily signed the informed consent forms, including consent for publication of their photographs. All of them were asked to accept any other treatment during the whole process. The study was approved by the Medical Ethics and Human Research Committee of China Medical University (approval number: 2023-571-2), and obeyed the principles of the 1975 Declaration of Helsinki. The study was registered in National Health Security Information Platform before enrollment (Register number: ChiCTR2300078863).

### Study design

The study followed the STROBE guidelines. Each participant’s face was randomly assigned as treatment side and control side from the midline by random number table, and washed with a neutral cleanser prior to treatment. The target areas (forehead, cheek and chin) on both sides were anesthetized with 5% topical compound lidocaine cream (Tongfang Pharmaceutical Group Co., Ltd, Beijing, China). Forty minutes later, the cream was wiped off. Two different FMR devices were used separately. The treatment side was treated with a high frequency electrocautery therapy equipment (Peninsula Timerevert Needle ™, United Ⅱ, Shenzhen Peninsula Medical Co., Ltd, China). The treating parameters were as follows, forehead: 60–150 ms, 6–10 W, 0.6–1.0 mm; cheek & chin: 60–150 ms, 6–10 W, 1.2–2.0 mm. The control side was treated high frequency skin treatment equipment (EndyMed ™, EndyMed Pro, The 3Deep Company, Israel). The treating parameters were as follows, forehead: 80 ms, 10 W, 1.5 mm; cheek & chin: 110 ms, 14 W, 2.5 mm. The pulse width, power and depth varied according to the participants’ tolerance to FMR, and the severity and location of the lesions. The needles of both devices were non-insulated. After treatment, recombinant human epidermal growth factor (RhEGF) gel (Guilin Pavay Gene Pharmaceutical Co. Ltd., Guilin, China)was topically smeared on the treatment areas twice daily for 1 week. All participants were instructed to avoid washing face for 48 h post-treatment.

Clinical photographs were obtained by a Visia facial imaging device (Canfield Scientific, Inc., Parsippany, NJ, USA) and a digital camera (EOS60D, Canon, Tokyo, Japan) and at baseline and at 1 day, 7 days, 1 month and 3 months after the treatment. The melanin index (MI) and erythema index (EI) were measured by Mexameter (MX18, Courage and Khazaka, Electronic GmbH, Cologne, Germany), stratum corneum hydration (SCH) and sebum of those areas were measured by Corneometer and Sebumeter (CM825, SM810, Courage and Khazaka, Monaderm), and dermal density was calculated by DUB skinscanner v5.18 (taberna pro medicum, Germany). Average value of all index on forehead, cheek and chin of both sides were calculated by three repeated measurements at each observation time.

### Efficacy evaluation

The treatment duration on each side was recorded independently by a third person besides the dermatologist and the participant. Treatment efficacy was evaluated by using participant’s satisfaction score and physician’s global assessment (PGA) score at 7 days, 1 month and 3 months after the treatment. PGA score was performed by 2 independent dermatologists, and asked for help for a third one with disagreement. The scores ranged from 0 to 10, where 0 indicated no satisfaction and 10 indicated complete satisfaction [16].

Pain score was evaluated by the participant and the bleeding score, erythema score was evaluated by the dermatology ranging from 0 to 10 on treatment side and control side, separately, with 0 indicating no pain or bleeding, and 10 indicating unbearable pain or severe bleeding. Any other side effects, including papules, exudation, desquamation, hyperpigmentation, hypopigmentation and scar, were reported by the participants and recorded by the dermatologists during and after the study.

### Statistical analysis

Statistical Package for Social Sciences (SPSS) Version 27.0.0.0 (IBM Corporation, Armonk, NY, USA) were used for statistical analysis. Quantitative data were shown as means ± standard deviation (SD). Comparisons between different groups were analyzed by paired-samples t-test, while those comparisons at different time points were performed by repeated-measure analysis of variance (ANOVA). *P* < 0.05 was considered as statistically significant.

## Results

### Basic information

Twenty participants (14 women and 6 men) completed the full course. Mean age was 30.40 ± 7.47 years (20–46 years). Five of them were diagnosed with photoaging (39.40 ± 6.27 years, 33–46 years), and 15 were diagnosed with atrophic acne scar (27.40 ± 5.10 years, 20–35 years). Treatment side of all participants were equal distributed on both sides, and vice versa.

### Efficacy evaluation

**Fig. 1:**
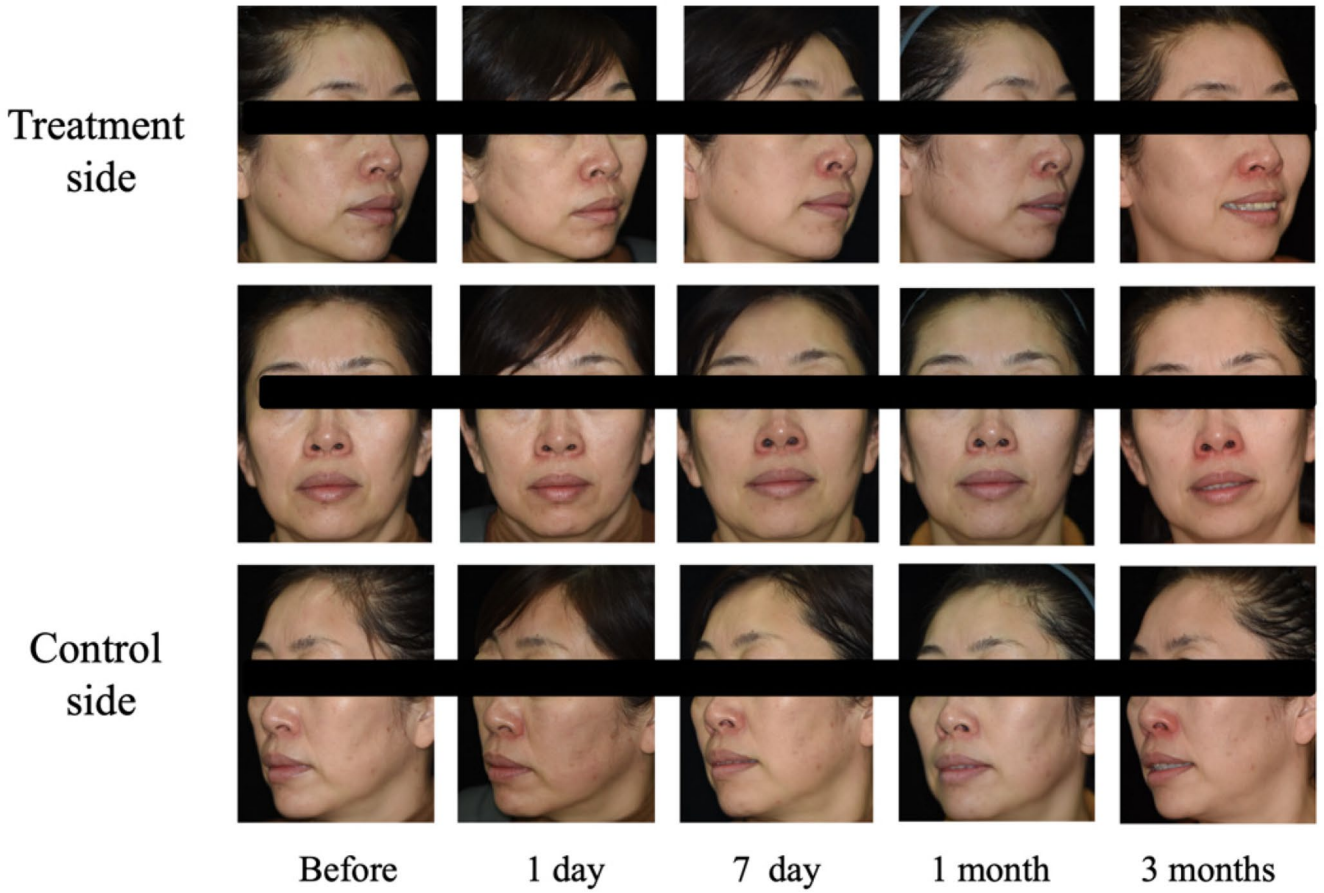
Clinical photos of a representative participant with photoaging

**Fig. 2:**
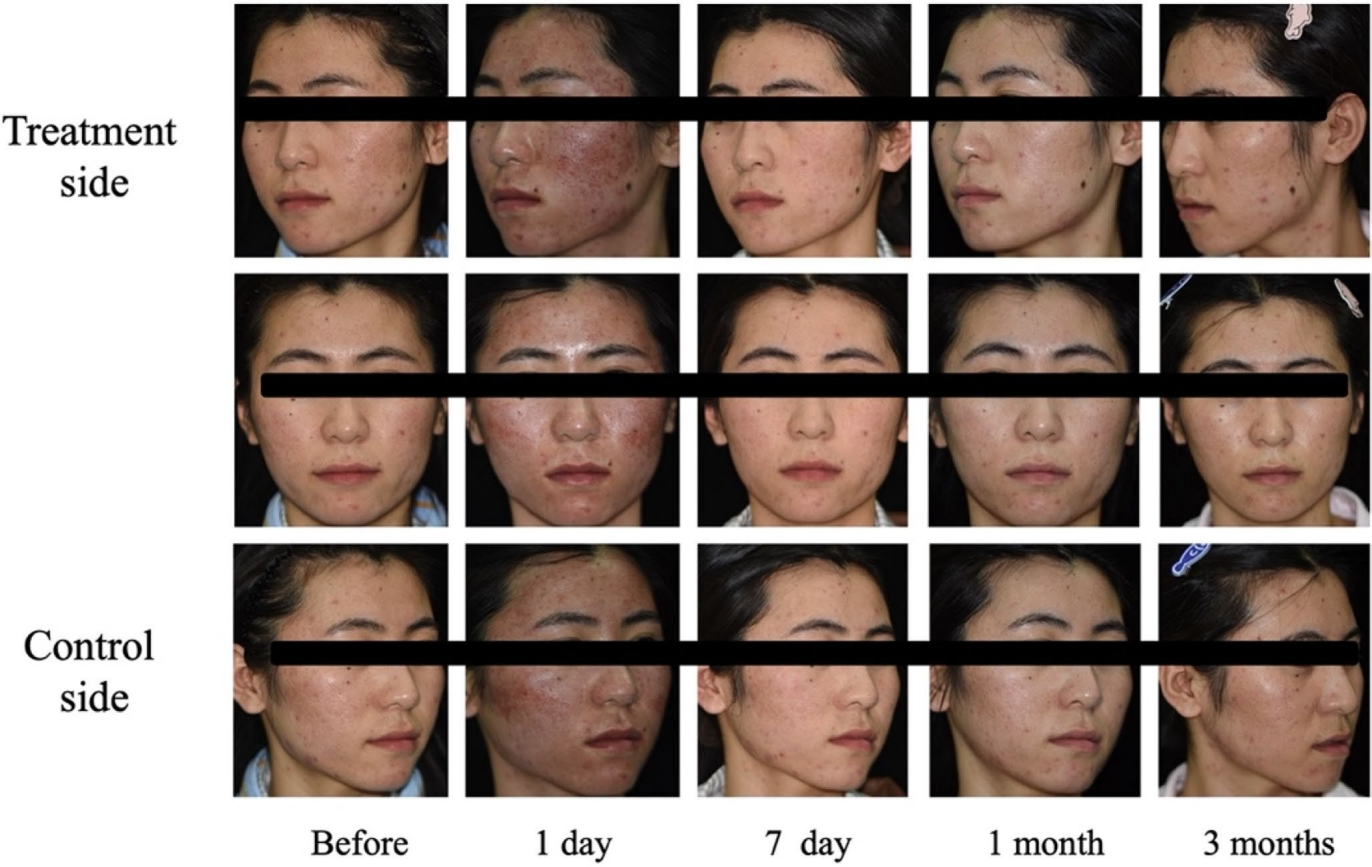
Clinical photos of a representative participant with acne scar

The efficacy of the FMR treatment improved over time. Participant’s satisfaction score on the treatment side increased from 1.85 ± 0.99 at Day 7 to 2.1 ± 1.12 at Month 1 and 2.78 ± 1.35 at Month 3, while the score on the control side increased from 1.90 ± 1.07 at Day 7 to 2.05 ± 1.15 at Month 1 and 2.67 ± 1.33 at Month 3. The differences between the two sides at all observation times were not statistically significant (*P* > 0.05). PGA score was in accordance with participant’s satisfaction score, with the highest value at Month 3 (3.18 ± 1.31 vs. 3.24 ± 1.03), but no statistical significances were seen between the two sides (*P* > 0.05). The clinical photos of representative participants diagnosed with photoaging was shown in Fig. 1, while those diagnosed with acne scar was shown in Fig. 2.

The treatment duration on treatment side (7.69 ± 2.76 min) was shorter than that on control side (10.21 ± 3.01 min) (*P* = 0.002). The pain score of treatment side (2.72 ± 1.86) was lower than that of control side (5.00 ± 1.94) (*P* = 0.000), but the bleeding score of treatment side (2.92 ± 1.91) was higher than that of control side (1.54 ± 1.68) (*P* = 0.000). The differences between treatment side and control side in erythema score (4.15 ± 2.01 vs. 4.00 ± 1.84, *P* = 0.732), erythema duration (30.20 ± 28.64 vs. 30.55 ± 27.61 h, *P* = 0.833) and edema duration (5.65 ± 11.90 vs. 6.65 ± 12.69 h, *P* = 0.552) were not statistically significant. One participant complained perioral blister, and another one reported dry skin. All of the above side effects were slightly on both sides, and recovered spontaneously in a few days without any treatment. No papule, exudation, hypopigmentation, desquamation and scar were found on both sides.

**Fig. 3:**
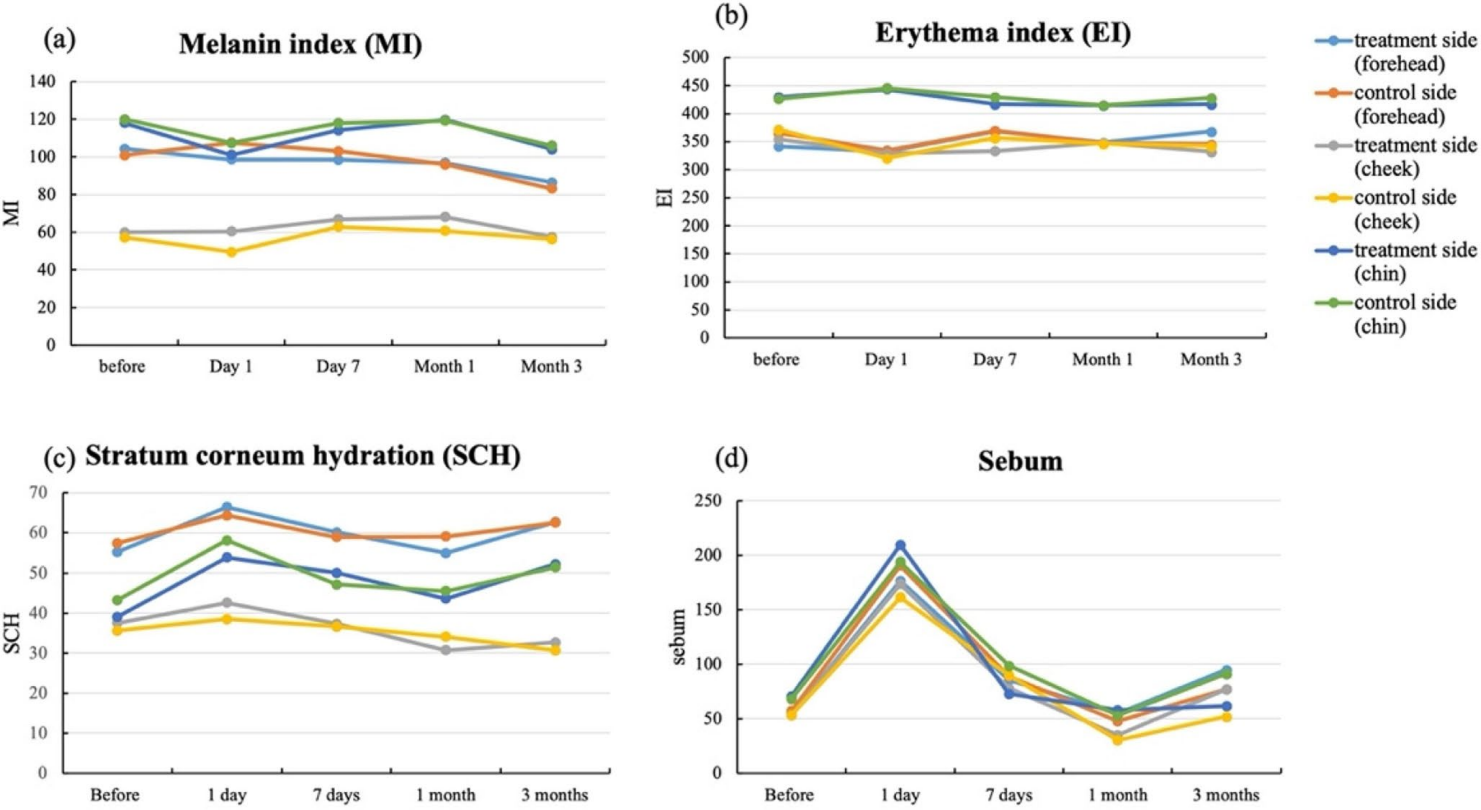
Comparisons between treatment side and control side on forehead, cheek and chin. (a) melanin index; (b) erythema index; (c) stratum corneum hydration; (d) sebum. All comparison between the two sides at different locations on all observation time weren’t statistically significant (*P* > 0.05). The values indicated averages of 20 participants

There were no statistical significances of MI, EI between forehead, cheek and chin of both sides at all observation times (*P* > 0.05, Fig. 3a and b). SCH and sebum increased 1 day after treatment but decreased to normal level at Day 7 on both sides of the 3 location (*P* > 0.05, Fig. 3c and d), which might be related to no face washing in the first 48 h after treatment.

**Fig. 4:**
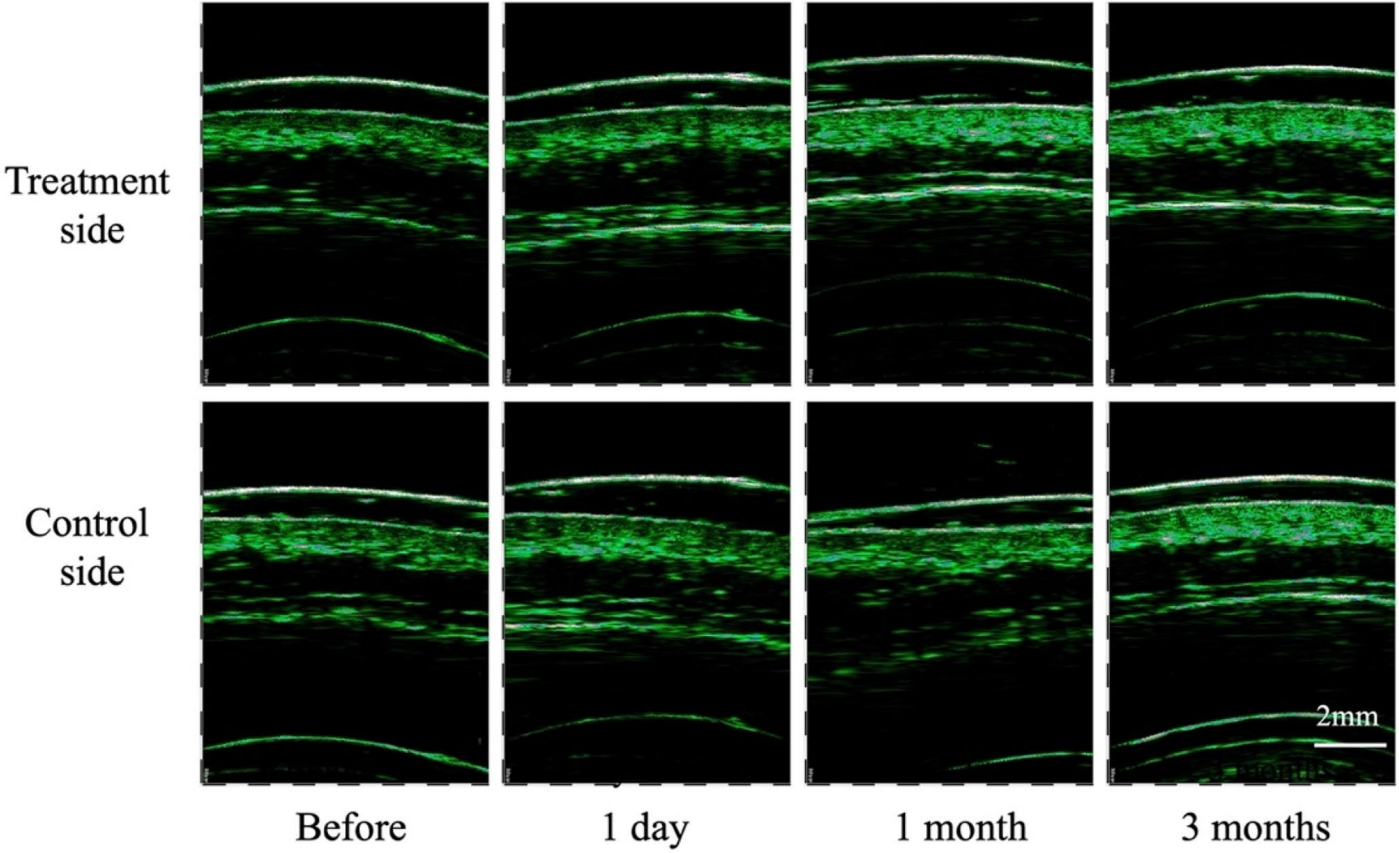
Dermal density of a representative participant examined by DUB skinscanner v5.18

The dermal density increased gradually after FMR treatment either in forehead, cheek and chin of treatment side or in those of control side, and was statistically significant at 3 months comparing with that before, 1 day and 7 days after treatment (*P* < 0.05). However, they didn’t show any statistical significance between the two sides (Fig. 4; Table 1). For the comparisons between dermis thickness, there weren’t statistical significances between treatment side and control side at all observation times (*P* > 0.05).

**Table 1:** Comparisons of dermal density between treatment side and control side

	Forehead		Cheek		Chin	
	Treatment side	Control side	Treatment side	Control side	Treatment side	Control side
Before	25.29 ± 9.38	9.38 ± 15	25.29 ± 9.38	25.58 ± 9.22	24.41 ± 9.09	23.7 ± 9.55
1 day	25.85 ± 13.51	13.51 ± 15	25.85 ± 13.51	27.57 ± 8.83	22.61 ± 10.97	23.13 ± 6.41
7 days	23.7 ± 11.82	24.59 ± 9.09	23.7 ± 11.82	24.59 ± 9.09	22.87 ± 9.96	23.84 ± 11.69
1 month	28.92 ± 12.86	28.94 ± 11.37	28.92 ± 12.86	28.94 ± 11.37	26.55 ± 12.16	24.69 ± 11.45
3 months	31.8 ± 10.36 ^a, b,c^	33.15 ± 12.41 ^a, b,c^	31.8 ± 10.36 ^a, b,c^	33.15 ± 12.41 ^a, b,c^	29.93 ± 11.85 ^a, b,c^	28.47 ± 9.4 ^a, b,c^

^a, b, c^ indicated *P*<0.05 comparing with dermal density before treatment, 1 day or 7 days after treatment, separately. The comparisons were conducted by repeated−measure analysis of variance (ANOVA)

## Discussion

FMR has been widely applied on various dermatologic conditions in the recent decade, such as skin rejuvenation, acne scars, acne vulgaris, striae and axillary hyperhidrosis, melasma, rosacea, cellulite, and androgenetic alopecia [17]. However, the mechanisms are still underestimated. In the present study, both participant’s satisfaction score and PGA score increased since Day 7, with the highest score 3 months after either FMR treatment, indicating excellent efficacy by both devices. The potential mechanism might be that RF restores the elasticity by stimulating collagen and elastin remodeling [18]. It increases the production of collagen fiber through thermal mechanisms and enhances heat shock protein (HSP47 and HSP90) expression. It also activates an ion channel named as Piezo1, which is implicated in macrophage polarization toward an M2 phenotype. Moreover, RF enhances TGF-β production and stimulates dermal–epidermal junction (DEJ) protein expression, which accelerates the production of collagen XVII [19]. Histological analysis in normal skin tissue of porcine models showed that FMR improved dermal coagulation with intervening zones, accompanied with a fractional pattern of epidermal ablation [20]. In our study, dermal density increased after FMR treatment, and the difference was statistically significant after 3 months compared with that at baseline. The result indicated that collagen and elastin were remolded after FMR treatment.

For skin barrier function, we tested SCH and sebum content of the epidermis in the present study. We found that No significant changes were observed in SCH or sebum levels, indicating that low energy used in FMR lead to invisible damage to the epidermal barrier. The result was in accordance with that of Ben Wang’s study. They applied non-insulated FMR on difficult-to-treat rosacea, and showed excellent improvement without destroying the skin barrier [21]. Although SCH and sebum increased temporary on the first day after FMR treatment in our study, it might be caused by no face washing in the first 48 h.

The efficacy of the 2 different FMR devices from different company were comparative. However, Peninsula Timerevert Needle ^TM^ showed less pain, shorter treatment time, but more bleeding comparing with EndyMed ™. The degree of erythema, erythema lasting time and edema lasting time were comparative.

Fractional carbon dioxide (fCO_2_) laser is excellent in improving skin textural irregularities, therefore, it is widely applied in skin rejuvenation [22] and acne scar [23]. But there are various side effects during and after fCO_2_ treatment, including erythema, edema, crust, pain and hyperpigmentation [24]. The efficacy of fCO_2_ laser and FMR are comparative in improving skin texture in acne scars. However, fCO_2_ laser caused more pronounced local skin reactions [25]. In our FMR study, no statistical significances were obtained in MI and EI on forehead, cheek and chin of both sides. These findings are consistent with previous reports. FMR doesn’t lead to less hyperpigmentation, for it delivers thermal energy to the dermis while sparing the epidermis [26]. Therefore, FMR is more acceptable by cosmetic patients with limited adverse effects.

In conclusion, FMR was effective and safe for skin photoaging and acne scar. Peninsula Timerevert Needle ^TM^ has the advantages of less pain and shorter treatment time, but more bleeding comparing with EndyMed ™. There were some limitations in this study. Only 20 participants were included in the present study and the observation time is limited. Therefore, more studies with larger sample size from multi-centers and more follow-ups are needed to verify the results.

## Data Availability

All data are available on request to corresponding author.

## Declarations

### Clinical trial registration

The study was registered in National Health Security Information Platform before enrollment (Register number: ChiCTR2300078863).
